# Genetic structure and diversity in Brazilian populations of *Anastrepha obliqua* (Diptera: Tephritidae)

**DOI:** 10.1371/journal.pone.0208997

**Published:** 2018-12-20

**Authors:** Joseane F. Passos, Danilo B. Nascimento, Rodolpho S. T. Menezes, Ricardo Adaime, Elton L. Araujo, Kátia M. Lima, Roberto A. Zucchi, Beatriz Ronchi Teles, Ruth R. Nascimento, Raul Ruiz Arce, Norman B. Barr, Bruce A. McPheron, Janisete G. Silva

**Affiliations:** 1 Departamento de Ciências Biológicas, Universidade Estadual de Santa Cruz. Ilhéus, Bahia, Brazil; 2 Departamento de Ciências Biológicas, Universidade Federal do Amapá. Macapá, Amapá, Brazil; 3 Departamento de Biologia, Faculdade de Filosofia, Ciências e Letras - Universidade de São Paulo, Ribeirão Preto, São Paulo, Brazil; 4 Empresa Brasileira de Pesquisa Agropecuária, Macapá, Amapá, Brazil; 5 Departamento de Ciências Biológicas. Universidade Federal Rural do Semi-Árido, Mossoró, Rio Grande do Norte, Brazil; 6 Departamento de Entomologia. Universidade de São Paulo, Piracicaba, São Paulo, Brazil; 7 Departamento de Entomologia. Instituto Nacional de Pesquisas da Amazônia, Manaus, Amazonas, Brazil; 8 Departamento de Ciências Biológicas. Universidade Federal de Alagoas, Maceió, Alagoas, Brazil; 9 USDA APHIS Science and Technology, Mission Laboratory, Edinburg, Texas, United States of America; 10 Department of Entomology, Ohio University, Columbus, Ohio, United States of America; National Cheng Kung University, TAIWAN

## Abstract

*Anastrepha obliqua* (Macquart), the West Indian fruit fly, is one of the most economically important pest species in the Neotropical region. It infests an extensive range of host plants that include over 60 species. The geographic range of *A*. *obliqua* is from northern Mexico to southern Brazil and includes the Caribbean Islands. Previous molecular studies have revealed significant genetic structure among populations. We used sequences from a fragment of the mitochondrial protein-coding gene cytochrome c oxidase I to estimate structure and genetic diversity of *A*. *obliqua* populations from Brazil. We analyzed a total of 153 specimens from the Amazon Forest, Atlantic Forest, Cerrado, and Caatinga biomes. Our study revealed weak genetic structure among the *A*. *obliqua* Brazilian populations sampled. Collections from the Amazon Forest had similar haplotype diversity compared to previously reported estimates for collections from the Caribbean and both populations are also closely related to each other, thus challenging the hypothesis that *A*. *obliqua* originated in the Caribbean and then moved to other regions of the Americas. Therefore, further evidence is necessary to draw a definite conclusion about the putative center of origin for *A*. *obliqua*. Additionally, we suggest a putative historical migration from the west to the east for the *A*. *obliqua* Brazilian populations, which could explain the high genetic diversity for this fly in the Amazon Forest and low genetic diversity in the other Brazilian biomes.

## Introduction

Tephritidae is one of the largest families of Diptera with over 4.500 species worldwide and includes some of the world’s most economically important fruit pests [1, 2]. The genus *Anastrepha* (Diptera: Tephritidae) is endemic to the Neotropical region where it is widespread and comprises around 296 species [3]. In Brazil, 120 species of *Anastrepha* have been reported infesting over 275 hosts in 48 plant families [4].

*Anastrepha obliqua* (Macquart), the West Indian fruit fly, is the second most polyphagous species within this genus in Brazil and therefore one of the most economically important pest species. It belongs within the *fraterculus* taxonomic group, which encompasses a total of 34 formally described species that can be distinguished only by morphological characters [2]. The aculeus and tip lengths of *A*. *obliqua* vary along a geographic cline and even from specimens reared from same host in Brazil [5]. This species ranges from northern Mexico to southern Brazil and also occurs in the Caribbean Islands [6]. It has been recorded occasionally in Florida, Texas, and California [6], all of which are outside its natural distribution [7]. It infests an extensive range of hosts, over 60 species from 24 plant families, with a strong preference for anacardiaceous species [6–8]. In Brazil, *A*. *obliqua* has been reported infesting 49 hosts [4].

Molecular studies using mitochondrial DNA (mtDNA) sequences revealed a high genetic diversity among *A*. *obliqua* populations [9,10]. Based on the cytochrome oxidase subunit I (COI) gene, for the phylogenetic reconstruction of the *fraterculus* group [9], eight *A*. *obliqua* individuals were clustered into two clades: (I) a clade containing Brazilian *A*. *obliqua*, *Anastrepha fraterculus* (Wiedemann), and *Anastrepha sororcula* specimens; and (II) a monophyletic lineage of four *A*. *obliqua* from Mexico, Brazil, and Colombia. Therefore, *A*. *obliqua* populations were not recovered as a monophyletic group raising the question of whether *A*. *obliqua* is a cryptic species complex.

A molecular study by Ruiz-Arce *et al*. [10] on the West Indian fruit fly sequenced two fragments of mtDNA genes [COI and NADH subunit six (ND6)] to test population genetic structure of collections from Mexico, Central America, the Caribbean, and eastern Brazil. The results revealed six distinct genetic clusters in the species that are strongly associated with geography. The genetic differences separating these clusters were similar to the divergence levels separating other species within the *fraterculus* group, suggesting there could be cryptic species in *A*. *obliqua*. The study also showed high haplotype diversity among specimens from the Caribbean and thus those authors raised the hypothesis that the Caribbean, most likely the Greater Antilles, could be the center of origin of *A*. *obliqua*. In a recent study, Scally *et al*. [11] analyzed intra-specific relationships within *A*. *obliqua* and interspecific relationships within the *fraterculus* group using a multi-locus data set (seven nuclear and two mitochondrial loci). The results from the nuclear loci support monophyly of this species, however, analysis of the mtDNA sequences showed high variability revealing mito-nuclear discordance in *A*. *obliqua*. Those authors suggested that mitochondrial introgression between *A*. *obliqua* and *A*. *fraterculus* is one possible explanation for this discrepancy and alternatively incomplete lineage sorting could have caused the high mitochondrial diversity observed in *A*. *obliqua*.

Variation among *A*. *obliqua* populations has also been documented in behavioral and ecological studies. In Mexico, *A*. *obliqua* matings took place in the early morning [12], contrasting with a study showing that *A*. *obliqua* populations in Brazil mated in the afternoon [13]. Additionally, morphometric studies have also detected divergence between populations of *A*. *obliqua* in Colombia, which formed two groups and probably more than one biological entity could be present within the nominal species [14].

The study by Ruiz-Arce *et al*. [10] detected population structure for relatively broad regions in the Americas but they did not investigate structure within Brazil because of limited fly collections. It is possible that *A*. *obliqua* populations are structured considering different Brazilian biomes. An in-depth knowledge of the genetic diversity and the population history of a species are important for pest management because it improves the capacity of action agencies and pest programs to predict how it will react to environmental changes [15, 16]. Here we sequenced a portion of the mitochondrial gene COI to evaluate the genetic structure and diversity as well as the historical demography of *A*. *obliqua* populations in four Brazilian biomes: Amazon Forest, Cerrado, Caatinga, and Atlantic Forest.

## Materials and methods

### Ethics statement

A permit for the collection of insects was provided by SISBIO (permanent permit number 30206–1 to RA). No collections involved endangered or protected species.

### Sampling, DNA extraction, amplification, and sequencing

**Fig 1:**
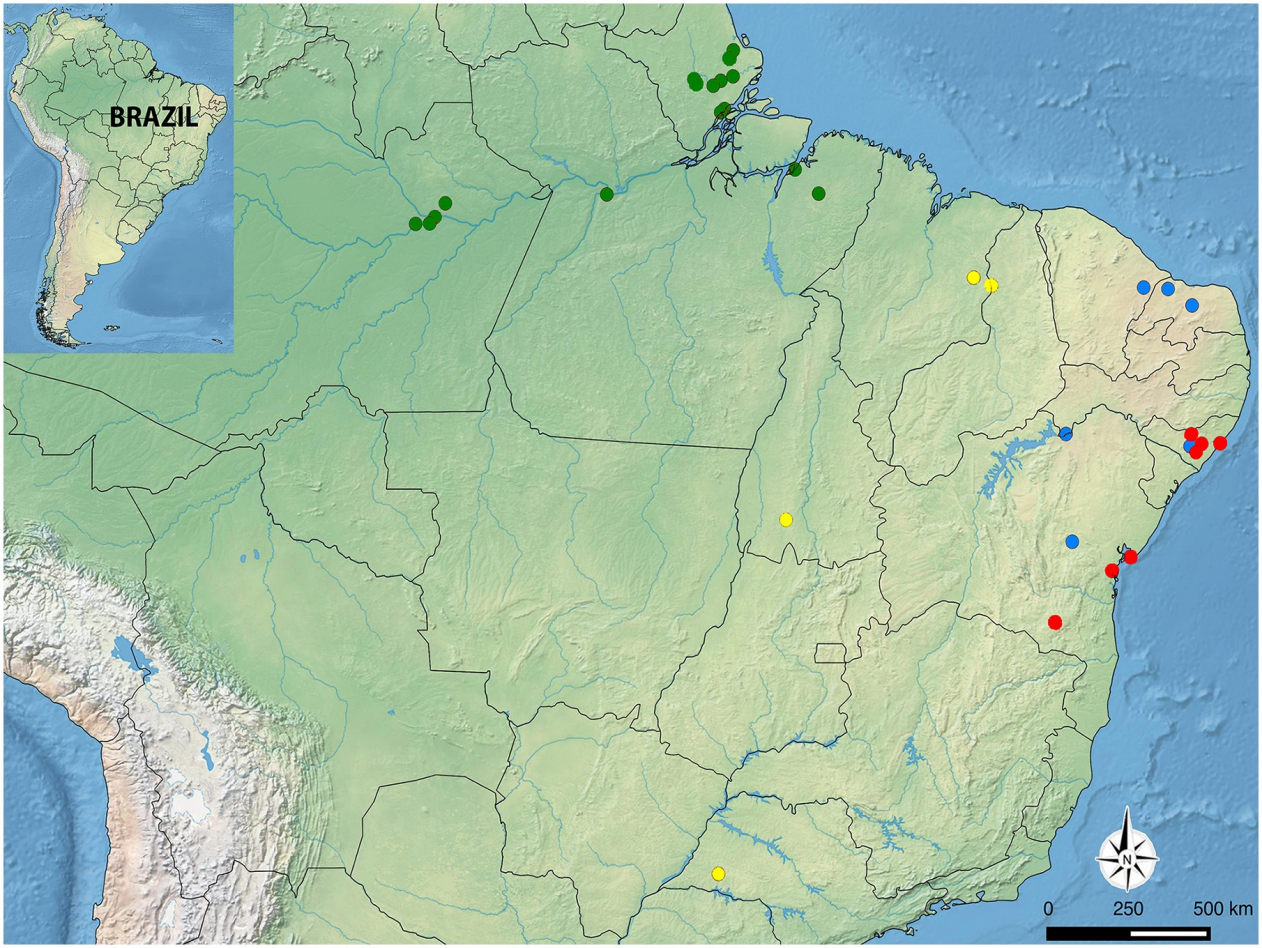
Map of northern areas in South America showing sampled localities and each dot represents a sampled locality from a specific Brazilian biome (Amazon Forest = green, Cerrado = yellow, Caatinga = blue, and Atlantic Forest = red).

*Anastrepha obliqua* samples were collected in 33 localities covering four distinct Brazilian biomes (Fig 1 and Table 1). Adults were either collected using McPhail traps or reared from ripe or ripening fruit collected randomly both from tree canopies and recently fallen. *Voucher* specimens were stored in 100% ethanol at -20°C prior to the molecular procedure. Total genomic DNA was isolated using the DNeasy Blood Tissue kit (Inc., Qiagen, Valencia, California, USA). A partial fragment of the *Cytochrome oxidase subunit* I (COI) was amplified by the Polymerase Chain Reaction (PCR) using primers: LCO-1490 (5-GGTCAACAAATCATAAAGATATTGG-3) and HCO-2198 (5-TAAACTTCAGGGTGACCAAAAAATCA-3) [17]. PCR was performed in 25 μl reactions containing 3.0 μl of 2.5 mM of MgCl_2_, 2.5 μl of 1 mM of dNTPs, 1.0 μl of 20 μM of each *primer*, 2 U of *Taq DNA polymerase* (Fermentas), and 2 μl of DNA. Cycling conditions for amplification were 3 min at 94 °C, followed by 39 cycles of 1 min at 94 °C, 1 min at 50 °C and 2 min at 72 °C, and a final extension step of 10 min at 72 °C. PCR products were purified using exonuclease I of *Escherichia coli* (EXOI) and shrimp alkaline phosphatase (SAP) (Fermentas/Thermo). Bi-directional sequencing was performed using the BigDye Terminator v3.1 Cycle Sequencing kit on an ABI 3730 of Life Technologies–Applied Biosystems at the Centro de Estudos do Genoma Humano, Universidade de São Paulo, Brazil. The forward and reverse sequences were imported to BIOEDIT v7.0.5.2 [18] to produce a consensus sequence for each sample and multiple sequence alignment was generated using Clustal W. All edited sequences were deposited at GenBank (accession numbers KY996561 –KY996713).

**Table 1:** Information regarding *Anastrepha obliqua* samples used in this study.

Biome/Group	Locality	Code	N	Host	Coordinates
AM	Manaus	AM1	10	*Eugenia stipitata*	-3°06’S, 60° 01’W
AM	Iranduba	AM2	2	*Averrhoa carambola*	3° 12’S, 60° 10’W
AM	Rio Preto da Eva	AM3	5	*E*. *stipitata*	2° 41’S, 59° 42’W
AM	Manacapuru	AM4	1	*Psidium guajava*	3° 17’S, 60° 38’W
AM	Serra do Navio	AP1	6	*Spondias mombin*	0° 51’N, 51° 11’W
AM	Macapá	AP2	9	*S*. *mombin*	0° 57’N, 50° 46’W
AM	Cutias	AP3	9	*S*. *mombin*	1° 43’N, 51° 05’W
AM	Santana	AP4	8	*S*. *mombin*	0° 47’N, 51° 56’W
AM	Tartarugalzinho	AP5	2	*S*. *mombin*	0° 02’N, 51° 03’W
AM	Pedra Branca	AP6	1	*S*. *mombin*	0° 52’N, 52° 01’W
AM	Ferreira Gomes	AP7	3	*S*. *mombin*	0° 46’N, 51° 56’W
AM	Porto Grande	AP8	2	*S*. *mombin*	1°30’N, 50°54’W
AM	Pracuuba	AP9	2	*S*. *mombin*	0° 02’N, 50° 46’W
AM	Santarém	PA1	3	Trap	2° 26’S, 54° 41’W
AM	Tomé-Açu	PA2	3	*S*. *mombin*	2° 24’S, 48° 08’W
AM	Ilha de Marajó	PA3	4	Trap	0° 59’N, 49° 35’W
CE	Cariri do Tocantins	TO1	5	*Mangifera indica*	11° 53’S, 49° 09’W
CE	Indiana	SP1	5	*P*. *guajava*	22° 10’S, 51° 15’W
CE	Caxias	MA1	5	*S*. *mombin*	4° 52’S, 43° 20’W
CE	Teresina	PI1	2	*S*. *mombin*	5° 05’S, 42° 48’W
CA	Limoeiro do Norte	CE1	10	*Spondias lutea*	5° 08’S, 38° 05’W
CA	Angicos	RN1	9	*S*. *lutea*	5° 40’S, 36° 36’W
CA	Mossoró	RN2	8	*S*. *lutea*	5° 11’S, 37° 20’W
CA	Petrolina	PE1	5	Trap	9° 23’S, 40° 30’W
CA	Itaberaba	BA1	2	Trap	12° 30’S, 40° 18’W
CA	Arapiraca	AL1	6	*Spondias*. *tuberosa*	9° 45’S, 36° 39’W
AF	Palmeira dos Índios	AL2	6	*Spondias purpurea*	9° 24’S, 36° 37’W
AF	Anadia	AL3	1	*A*. *carambola*	9° 41’S, 36° 18’W
AF	Maceió	AL4	5	*S*. *mombin*	9° 39’S, 35° 44’W
AF	Junqueiro	AL5	2	*A*. *carambola*	9° 54’S, 36° 28’W
AF	Valença	BA2	2	*S*. *mombin*	9° 41’S, 36° 18’W
AF	Salvador	BA3	5	Trap	12° 58’S, 38° 30’W
AF	Vitória da Conquista	BA4	5	Trap	14° 51’S, 40° 50’W

Biome/Group: Amazon Forest (AM), Cerrado (CE), Caatinga (CA), and Atlantic Forest (AF). Codes represent the Brazilian states: Amazonas (AM), Amapá (AP), Pará (PA), Tocantins (TO), São Paulo (SP), Maranhão (MA), Piauí (PI), Ceará (CE), Rio Grande do Norte (RN), Pernambuco (PE), Bahia (BA), and Alagoas (AL). N: number of specimens collected.

### Population structure, phylogenetic relationships, and genetic diversity

**Fig 2:**
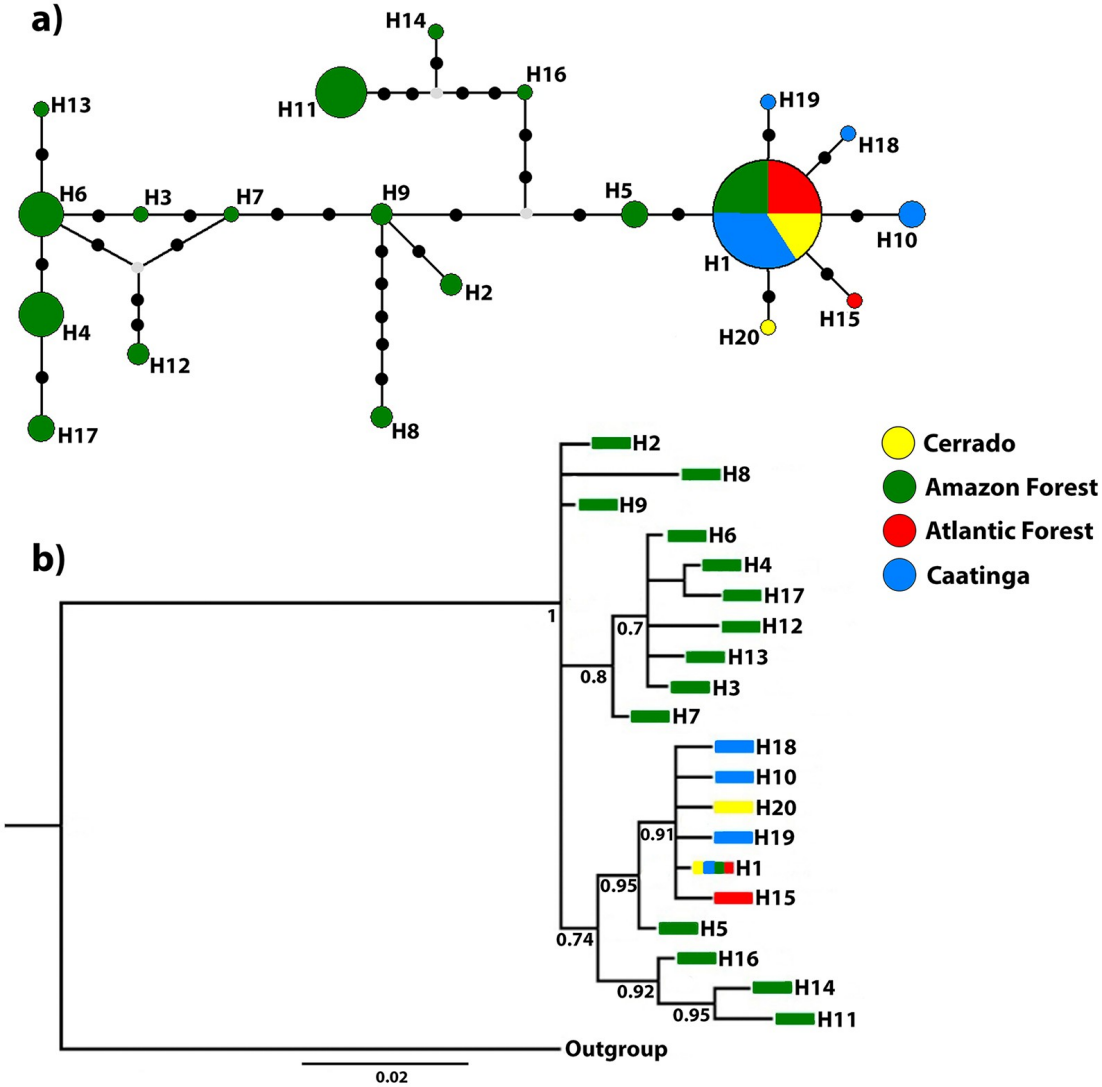
Population genetic structure. (a) Unrooted haplotype network based on 621 bp of COI. Each circle represents one haplotype and the size is proportional to its frequency among the samples. Small black dots represent mutational steps and small gray dots represent median vectors. The abbreviations are in the caption for (a). (b) Bayesian inference of *A*. *obliqua* haplotypes. Numbers below branches indicate posterior probabilities.

We initially delineated populations according to Brazilian biomes, as follows: Amazon Forest (AM), Cerrado (CE), Caatinga (CA), and the Atlantic Forest (AF). We constructed a haplotype network using median-joining method [19] in Network v4.603 (www.fluxus-engineering.com) to infer the relationships among haplotypes and their geographical distribution. We examined genetic structure among populations using F-statistics [20] and Analysis of Molecular Variance (AMOVA) in Arlequin v3.1 [21], considering two hierarchical levels. We also estimated two additional AMOVA analyses with three hierarchical levels each one. For the third level of AMOVA, we considered (1) four hypothetical groups according to Brazilian biomes (AM, CA, CE, and AF), and (2) separating the populations in two groups (AM versus CA, CE and AF) according to our phylogenetic and network analyses (see Fig 2). We evaluated the correlation between genetic and geographic distance among the populations using Mantel Tests [22] implemented in IBDWS v3.23 [23]. Bayesian inference (BI) was conducted using MrBayes v3.2.4 [24] to infer the phylogenetic relationships among haplotypes. Additionally, we performed Neighbor-Joining (NJ) and Maximum Likelihood (ML) analyses using MEGA v6 [25] with 1000 bootstraps each and using COI sequences from Caribbean flies previously published by Ruiz-Arce *et al*. [10]. The appropriate nucleotide substitution model (herein GTR+ Γ) was selected using JModeltest v3.7 [26]. The K_2_P model was used to calculate pairwise distances using MEGA v6 and Arlequin v3.1.

BI was carried out with two simultaneously runs of 10 million generations each with four chains and sampled every 1,000 generations. We used Tracer v1.6 [27] to examine run convergence through ESS values (> 200). The first 25% of the trees were discarded as burn-in and the remaining trees were used to produce the maximum credibility tree that was visualized and edited using FigTree v1.4.0 [28]. A sequence of *Anastrepha serpentina* (Wiedemann) obtained from the Genbank (GenBank: RX130130) was used as the outgroup.

Finally, we calculated the nucleotide diversity (π), number of haplotypes (h), haplotype diversity (Hd), and number of polymorphic/segregating sites (*S*) for the entire dataset and for each group using DNAsp v5.0 [29].

### Historical demography

Demographic history was inferred based on the neutrality tests and assumptions of constant population size using the Tajima’s *D* [30], Fu’s *F*_S_ [31] and R_2_ statistics [32] calculated in DNAsp v5.0. These tests were estimated with 10,000 coalescent simulations to calculate significance values and to test the hypotheses that all mutations are selectively neutral. We also estimated mismatch distributions [33] and calculated Sum of Squared Deviations (SSD) and Harpending’s raggedness index (rg) in Arlequin v3.1 [21]. Specifically, for these tests we assumed two groups, AM and the eastern portion of South America (CE, CA, and AF). We expected to detect signal of population expansion in the colonized region but not at the center of origin.

Additionally, we performed a Bayesian Skyline Plot (BSP) analysis [34] implemented in BEAST v1.8.2 (Bayesian Evolutionary Analysis Sampling Trees) [35] to estimate changes in effective population size over time. We implemented a strict molecular clock prior with a standard invertebrate mitochondrial divergence rate (1.5% per million years) [36], due to the absence of useful fossil calibrations in *Anastrepha*. We performed two replicate runs with 100 million of generations, with tree sampling every 10,000 generations. Tracer v1.6 [27] was used to verify convergence (ESS > 200). Replicate runs were combined after a burn-in of 20% using LogCombiner v1.8.2.

## Results

Our sequence data set comprised 621 base pairs (bp) of the mtDNA COI fragment from 153 specimens. The sequenced region contained 27 variable sites defining 20 different haplotypes. No premature stop codons or indels were found in the COI fragment. The most common haplotype (H1) was detected in 66% (n = 101) of the samples and it was observed over a widespread geographic range and among all geographic groups (CE, CA, AM, and AF). The collections from AM showed 15 haplotypes, out of which 14 were singletons. The CE, CA, and AF populations showed two, four and two haplotypes, respectively (Table 2). The average genetic distance varied between 0.2% and 1.6% among *A*. *obliqua* haplotypes (S1 Table).

**Table 2:** Distribution of 20 haplotypes (H) observed among the *Anastrepha obliqua* Brazilian collections in the biomes studied and GenBank access numbers. Collection site codes are according to geographic sites shown in Table 1.

Biome	H	Collection Site	GBAccess#
**AF**	H1	AL2 (6), AL3(1), AL4 (5), AL5(2) BA2 (2), BA3 (5), BA4 (4)	KY996561
	H15	BA4 (1)	KY996666
**CA**	H1	CE1(9), RN1(8), RN2(8), PE1(5), BA1(2), AL1(3)	KY996561
	H10	AL1(3)	KY996618
	H18	RN1(1)	KY996690
	H19	CE1(1)	KY996703
**CE**	H1	TO1(5), SP1 (4), MA1(5) PI1(2),	KY996561
	H20	SP1 (1)	KY996712
AM	H1	AP1(4), AP2(6), AP3(8), AP4(5), PA2(1)	KY996561
	H2	AP1(1), AP2(1)	KY996570
	H3	AP1(1)	KY996566
	H4	AP2(1), AP4(1), AP5(2), AM3(2), PA1(1), PA3(1)	KY996638
	H5	AP2(1), AP7(2)	KY996575
	H6	AP3(1), AP4(1), AP7(1), AM1(2), AM3(1), PA3(1)	KY996582
	H7	AP6(1)	KY996595
	H8	AP8(2)	KY996599
	H9	AP9(2)	KY996601
	H11	AM1(4), AM3(2), AM4(1), PA2(1)	KY996627
	H12	PA3(2)	KY996636
	H13	PA1(1)	KY996640
	H14	PA1(1)	KY996641
	H16	AM1(1)	KY996672
	H17	AM1(3)	KY996674

In parentheses the frequencies of each haplotype and respective codes

**Fig 3:**
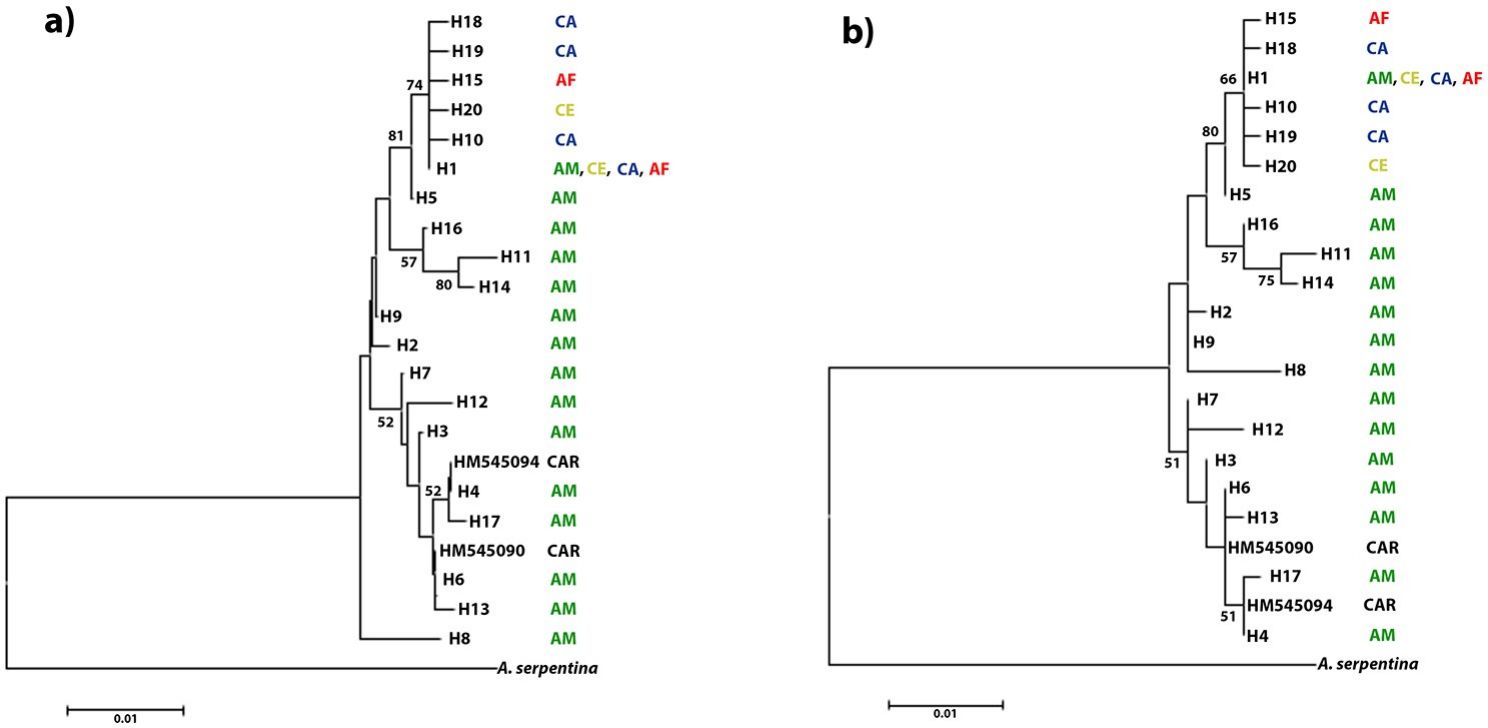
Phylogenetic analyses using *A*. *obliqua* specimens from the Caribbean and Brazilian regions. (a) Neighbor-Joining and (b) Maximum Likelihood trees. Numbers represent bootstrap support. CAR: Caribbean region, AM: Amazon Forest, AF: Atlantic Forest, CA: Caatinga, and CE: Cerrado.

Our unrooted haplotype network (Fig 2a) demonstrates that the Brazilian flies are genetically diverse with many haplotypes separated by multiple mutation steps. Although the eastern portion of South America (CE, CA, and AF) represented the majority of individuals analyzed (n = 83; 54.24% of our total sampling) they showed only six haplotypes, reflecting a low genetic diversity within these populations. When the flies from the Amazon Forest are excluded, the network exhibits a star-like topology with short branch lengths in which most of the unique haplotypes were closely related to the common central haplotype (H1) (data not shown). Our BI tree was congruent with the unrooted haplotype network (Fig 2b). Additionally, our NJ and ML analyses comprising Caribbean and Brazilian flies showed that the Caribbean haplotypes form a clade with haplotypes from northern Amazon region (Fig 3).

Our AMOVA test did not find any evidence for explicit physical barrier or host association triggering genetic structure in *A*. *obliqua* Brazilian populations (Tables 3 and 4). Our Mantel tests indicated a non-significant correlation between genetic and geographic distance (r = 0.0372, p = 0.2450).

**Table 3:** Partitioning of DNA variance as revealed by Analysis of Molecular Variance (AMOVA) based on two hierarchical levels for *Anastrepha obliqua* Brazilian populations.

Source of variation	Sum of squares	Variance components	Percentage of variation
Among populations	192.690	2.92240 Va	87.40
Within populations	63.218	0.42146 Vb	12.60

Fixation Index Fst: 0.87396, p<0.001.

**Table 4:** Partitioning of DNA variance as revealed by AMOVA based on three hierarchical levels for *Anastrepha obliqua* sampled in Brazil.

Groups			Among populations			
	Within populations		Within groups		Among groups	
	% Var	Fst	% Var	Fsc	% Var	Fct
**1. AM, FA, CE and CA (n = 4)**	42.96	0.57042*	28.88*	0.40204	28.16	0.28158
**2. AM vs CE+CA+AF (n = 2)**	59.46	0.40541*	-2.09	-0.03639	42.63	0.42629

p-values were calculated with 10,000 coalescent simulations.

*p<0.001.

**Fig 4:**
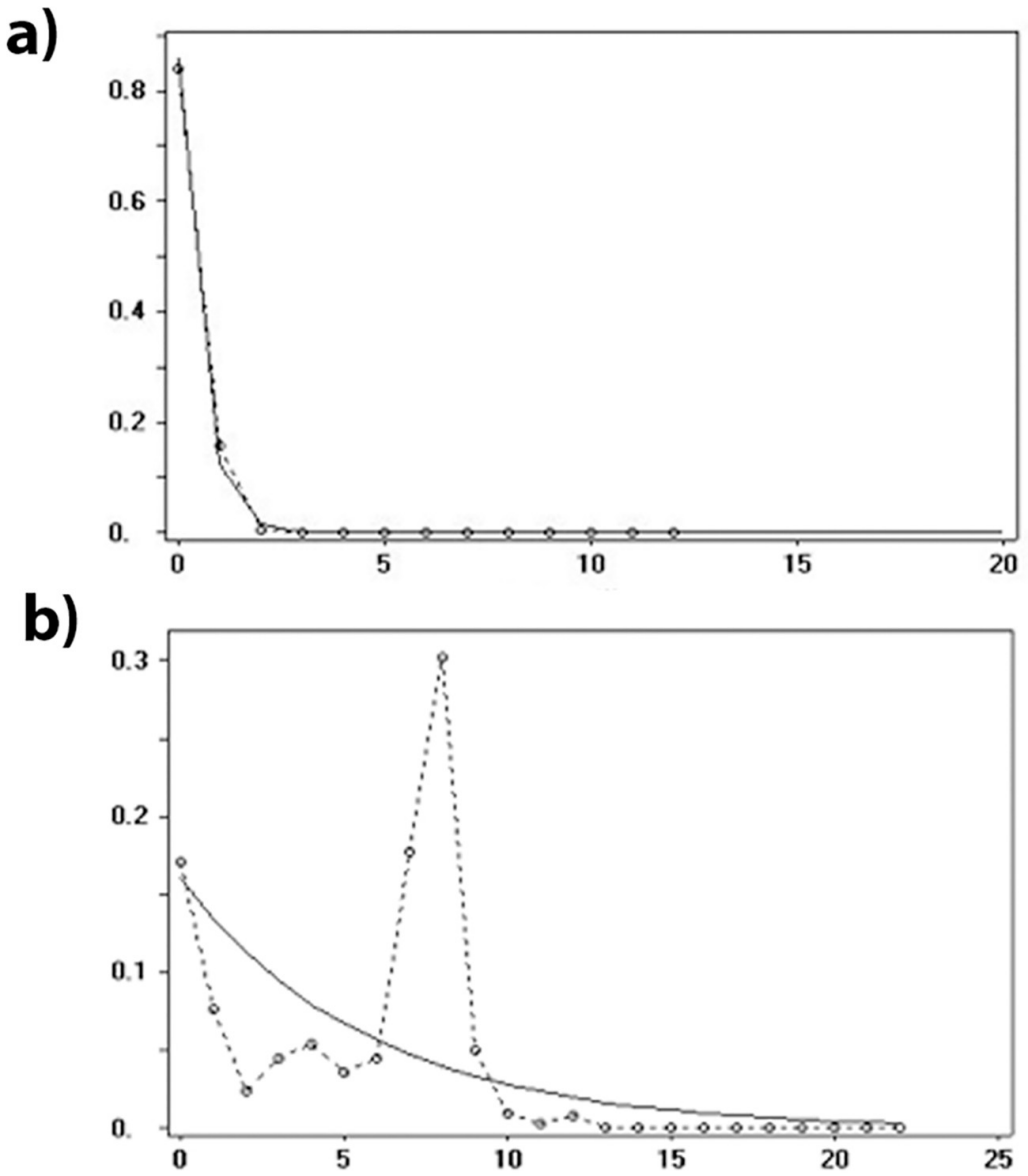
Mismatch distribution of the *Anastrepha obliqua* Brazilian populations. (A) AF+CE+CA group and (B) AM group. The expected frequency is based on a population growth-decline model determined using the DNAsp v.5.0 and is represented by a continuous line. The observed frequency is represented by a dotted line. The x axis shows the number of pairwise differences, the y axis shows the frequency of pairwise comparisons.

The genetic diversity scores inferred from COI for the entire dataset and for each group are shown in Table 5. The AM populations showed a higher genetic diversity (Hd = 0.8298 and π = 0.00847) when compared to the remaining groups (approximately a 8-fold increase). Neutrality and mismatch distribution tests were performed for the two biome groupings (AM and AF+CA+CE) separately as well as for the total *A*. *obliqua* data set. Neutrality tests revealed a genetic signature of demographic expansion when considering only populations from the eastern portion of South America (CE, CA, and AF) and unimodal curves that fit a sudden expansion model. Conversely, the mismatch distribution for the AM group showed a bimodal shape (Fig 4). The SSD and rH of group CE, CA, and AF showed estimates that were not significant, which indicate that the data have relatively good fit to a model of population expansion [37] (Table 5).

**Table 5:** Statistic summary for the COI analysis of *Anastrepha obliqua* populations.

Populations	n	*Hd* (±S.D.)	π (±S.D.)	*D*	*Fs*	*R*_*2*_	*SSD*	*rH*
AF+CA+CE	83	0.1616(0.055)	0.00027(0.00009)	**-1.83219**	**-7.01023**	0.04340	0.00065	0.48803
AM	70	0.8298(0.038)	0.00847(0.0045)	0.46575	-0.05912	0.11897	**0.07337**	**0.11037**
Total	153	0.556(0.048)	0.00542(0.00055)	-0.86391	-3.46712	0.06149	0.01913	0.44286

*h* = haplotype diversity, π = nucleotide diversity, *D* = Tajima’D, *Fs* = Fu’s Fs, SSD = Sum of Squared Deviations, rH—raggedness index, in bold statistically significant values, p<0.05.

**Fig 5:**
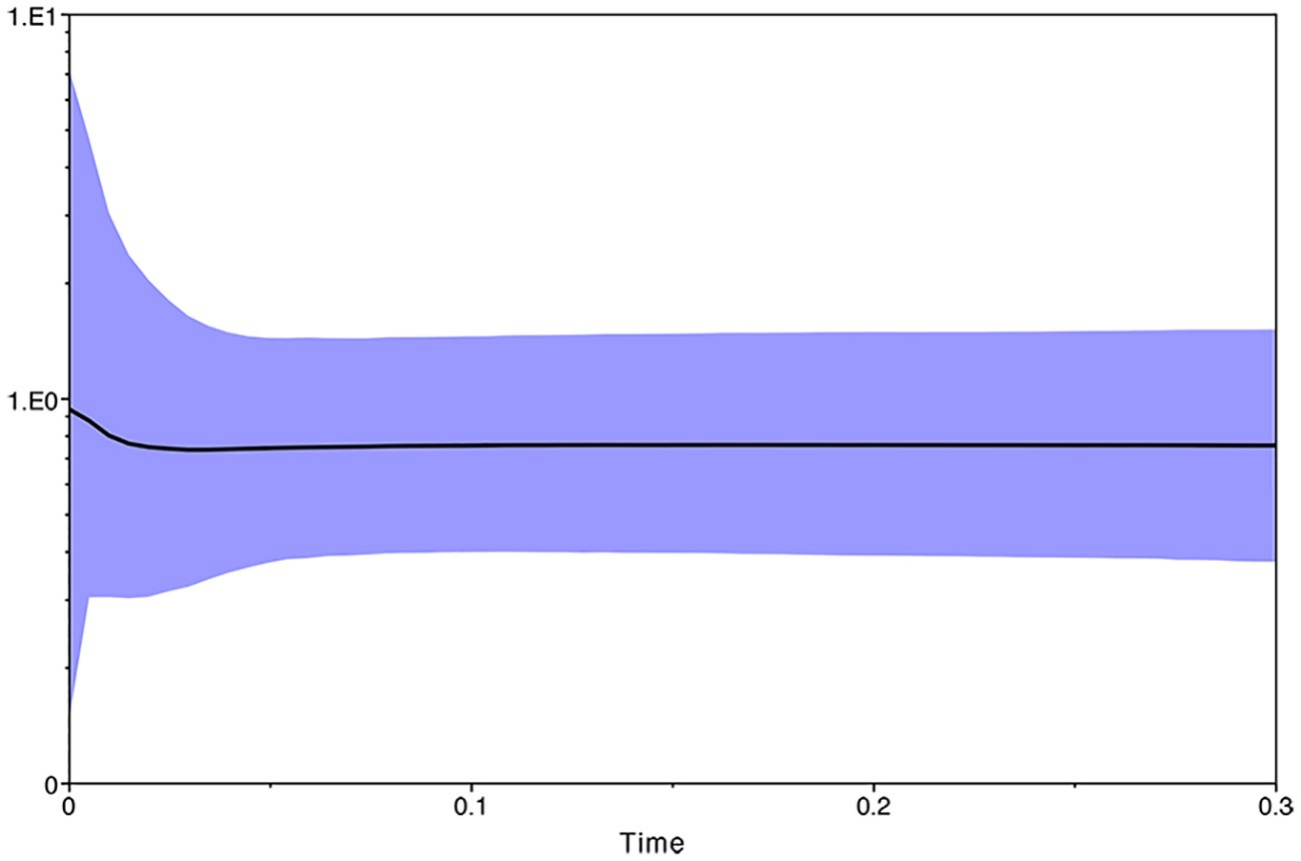
Bayesian skyline plot based on COI sequences of *Anastrepha obliqua* populations from Brazil. The median posterior estimates of demographic change over the past 300,000 years is graphed with the purple area indicating the 95% HPD. The Y-axis is in log scale. The X-axis represents time in millions of years (mya).

Our BSP analysis based on all groups showed demographic equilibrium (Fig 5) and recent and slight postglacial expansion starting approximately after the Last Glacial Maximum (LGM; 21,000 years ago).

## Discussion

### Population structure and genetic diversity

Previous molecular studies using mitochondrial DNA genes have shown a relatively high genetic diversity of the West Indian fruit fly [9,10]. Our study also revealed a high genetic diversity within Brazilian populations. However, our study used a distinct region of the COI gene than the one used in the Smith-Caldas *et al*. [9] study and smaller sequences than those used by Ruiz *et al*. [10], thus precluding direct comparison of diversity levels across studies. Ruiz-Arce *et al*. [10] found strong genetic structure in *A*. *obliqua* populations from Mesoamerica, western Mexico, Central America, the Andean and Caribbean regions, and eastern Brazil, suggesting the occurrence of some geographic barrier to gene flow. Our data did not detect population structure across the Brazilian biomes. In addition, no phylogeographic pattern was observed among Brazilian biomes other than low genetic diversity for biomes on the eastern portion of Brazil. The H1 haplotype is predominant in CE, CA, and AF biomes and present in the AM biome (specifically Amapá and Pará) indicating that there was gene exchange between the *A*. *obliqua* populations. Lack of population structure could be due to human-aided dispersal and/or that these populations cross naturally in the field.

The fly populations from the CE, CA, and AF biomes had low mtDNA haplotype and nucleotide diversity values, which could indicate that these populations have experienced a recent population expansion after a small effective population size or a bottleneck effect. Low haplotype and nucleotide diversity can indicate that demographic events have affected populations, such as a decline in population size or founding effect [38]. When the flies from the Amazon Forest are excluded from the data set, the haplotypes form a network exhibiting a star like topology with the haplotype H1 at the center.

Low levels of genetic diversity were detected in *A*. *obliqua* populations from Mesoamerica and Western Mexico [10]. Similarly, Lima 2011 [39] analyzed *A*. *obliqua* populations from Northeastern and Southeastern Brazil and also observed low genetic diversity using nuclear genes, which could be associated with a founder and bottleneck events followed by a recent population expansion, thus corroborating our results on *A*. *obliqua* populations from eastern Brazil (CA, CE and AF populations).

Considering our sampling, the populations from the Amazon region showed the highest levels of haplotype (Hd = 0.8298) and nucleotide (π = 0.00847) diversity. These populations had several private haplotypes, which could have resulted from limited migration and gene flow among them. Ancestral populations generally show high levels of genetic diversity in contrast with populations established more recently [40]. Moreover, it has recently been proposed that the geographic regions with populations showing high levels of genetic diversity acted as dispersal centers for the fruit flies *Bactrocera dorsalis* (Hendel) and *Anastrepha ludens* (Loew) [41,42]. Based on our current sampling for the Amazon region, we see a pattern of high haplotype diversity (Hd = 0.814), which is very similar to what was observed in populations from the Caribbean, considered as the probable center of origin for *A*. *obliqua* [10]. Interestingly, Caribbean haplotypes are closely related to haplotypes from the northern Amazon region suggesting a connection between these two geographic regions (Fig 3). Some of the sequences seen among Caribbean samples are not exclusive to that region as they are shared with others, including Mesoamerica [10]. Both the Caribbean and Amazon represent regions of high diversity for this fly that arose independently from an ancestral source. Thus, further evidence is necessary to draw a definite conclusion about the putative center of origin for *A*. *obliqua*. Resolving the origin and pathway for these sequence types may require additional and unlinked markers. With respect to collections gathered from the biomes included in our study, the haplotypes diversity estimates suggest the Amazon Forest is probably the source of this species in this region.

### Demographic history

The negative values of neutrality tests and unimodal mismatch distribution suggest that the populations from eastern South America (CA+CE+AF) have undergone a recent demographic expansion. In contrast, the AM group exhibited multimodal mismatch distribution patterns suggesting demographic equilibrium or a stable population, indicating that these populations have been stable over time. Thus, we detected signals of demographic expansion in the East (CE, CA and AF) but not in the West (AM) for *A*. *obliqua* Brazilian populations. Long-distance dispersal events tend to result in reduced genetic diversity due to sequential founding events and genetic drift [43]. The tests about demographic expansion performed by Ruiz-Arce *et al*. [10] for the Caribbean *A*. *obliqua* populations were incongruent since the mismatch distribution was unimodal and consistent with a population expansion event, but the neutrality tests were not significant. Hence, based only on the demographic tests both scenarios on the origin of *A*. *obliqua* (Caribbean versus Amazon) are equally parsimonious.

Our BSP analysis revealed stability followed by a recent population expansion in the *A*. *obliqua* Brazilian populations. Similarly, Ruiz-Arce *et al*. [10] verified signs of demographic expansion in *A*. *obliqua* populations from Mesoamerica, suggesting a bottleneck event followed by expansion. Changes in the geographic and climatic patterns over time might be associated with the distribution of genetic variability in *A*. *obliqua* populations from South America.

As our results are based solely on mtDNA sequence data, they should be viewed with some caution, since our conclusions might be biased due the intrinsic mutation rate, mode of inheritance, and effective population size of mtDNA markers [44]. Additionally, there is evidence of introgression between *A*. *obliqua* and *A*. *fraterculus* [11] and that may also be present among our sampling. Thus, further studies integrating ecological, morphological, and molecular (using mtDNA and nuclear markers) data are necessary to understand the evolutionary history of the West Indian fruit fly.

## Supporting information

S1 Table Estimate of genetic distance of *Anastrepha obliqua* haplotypes based on sequencing of a fragment of the mitochondrial COI gene. Analyses were conducted using the K_2_P model.
(DOCX)
