# Supplementary material for: Genetic structure and diversity in Brazilian populations of Anastrepha obliqua (Diptera: Tephritidae)
Source: PLoS One. 2018 Dec 20;13(12):e0208997. doi: 10.1371/journal.pone.0208997 (PMC6301665; doi:10.1371/journal.pone.0208997)
Supplement: S1 Table — Analyses were conducted using the K2P model. (DOCX) [file pone.0208997.s001.docx]

**S1 Table. Estimate of genetic distance** **of *Anastrepha obliqua* haplotypes** based on sequencing of a fragment of the mitochondrial COI gene. Analyses were conducted using the K_2_P model.

|  | **H1** | **H2** | **H3** | **H4** | **H5** | **H6** | **H7** | **H8** | **H9** | **H10** | **H11** | **H12** | **H13** | **H14** | **H15** | **H16** | **H17** | **H18** | **H19** | **H20** |
| --- | --- | --- | --- | --- | --- | --- | --- | --- | --- | --- | --- | --- | --- | --- | --- | --- | --- | --- | --- | --- |
| **H1** |  |  |  |  |  |  |  |  |  |  |  |  |  |  |  |  |  |  |  |  |
| **H2** | 0.006 |  |  |  |  |  |  |  |  |  |  |  |  |  |  |  |  |  |  |  |
| **H3** | 0.010 | 0.006 |  |  |  |  |  |  |  |  |  |  |  |  |  |  |  |  |  |  |
| **H4** | 0.013 | 0.010 | 0.003 |  |  |  |  |  |  |  |  |  |  |  |  |  |  |  |  |  |
| **H5** | 0.002 | 0.005 | 0.008 | 0.011 |  |  |  |  |  |  |  |  |  |  |  |  |  |  |  |  |
| **H6** | 0.011 | 0.008 | 0.002 | 0.002 | 0.010 |  |  |  |  |  |  |  |  |  |  |  |  |  |  |  |
| **H7** | 0.008 | 0.005 | 0.002 | 0.005 | 0.006 | 0.003 |  |  |  |  |  |  |  |  |  |  |  |  |  |  |
| **H8** | 0.013 | 0.010 | 0.013 | 0.016 | 0.011 | 0.015 | 0.011 |  |  |  |  |  |  |  |  |  |  |  |  |  |
| **H9** | 0.005 | 0.002 | 0.005 | 0.008 | 0.003 | 0.006 | 0.003 | 0.008 |  |  |  |  |  |  |  |  |  |  |  |  |
| **H10** | 0.002 | 0.008 | 0.011 | 0.015 | 0.003 | 0.013 | 0.010 | 0.015 | 0.006 |  |  |  |  |  |  |  |  |  |  |  |
| **H11** | 0.013 | 0.013 | 0.013 | 0.013 | 0.011 | 0.011 | 0.011 | 0.020 | 0.011 | 0.015 |  |  |  |  |  |  |  |  |  |  |
| **H12** | 0.013 | 0.010 | 0.006 | 0.006 | 0.011 | 0.005 | 0.005 | 0.016 | 0.008 | 0.015 | 0.013 |  |  |  |  |  |  |  |  |  |
| **H13** | 0.013 | 0.010 | 0.003 | 0.003 | 0.011 | 0.002 | 0.005 | 0.016 | 0.008 | 0.015 | 0.013 | 0.006 |  |  |  |  |  |  |  |  |
| **H14** | 0.011 | 0.011 | 0.011 | 0.011 | 0.010 | 0.010 | 0.010 | 0.018 | 0.010 | 0.013 | 0.005 | 0.011 | 0.011 |  |  |  |  |  |  |  |
| **H15** | 0.002 | 0.008 | 0.011 | 0.015 | 0.003 | 0.013 | 0.010 | 0.015 | 0.006 | 0.003 | 0.015 | 0.015 | 0.015 | 0.013 |  |  |  |  |  |  |
| **H16** | 0.006 | 0.006 | 0.010 | 0.010 | 0.005 | 0.008 | 0.008 | 0.013 | 0.005 | 0.008 | 0.006 | 0.010 | 0.010 | 0.005 | 0.008 |  |  |  |  |  |
| **H17** | 0.015 | 0.011 | 0.005 | 0.002 | 0.013 | 0.003 | 0.006 | 0.018 | 0.010 | 0.016 | 0.015 | 0.008 | 0.005 | 0.010 | 0.016 | 0.011 |  |  |  |  |
| **H18** | 0.002 | 0.008 | 0.011 | 0.015 | 0.003 | 0.013 | 0.010 | 0.015 | 0.006 | 0.003 | 0.015 | 0.015 | 0.015 | 0.013 | 0.003 | 0.008 | 0.016 |  |  |  |
| **H19** | 0.002 | 0.008 | 0.011 | 0.015 | 0.003 | 0.013 | 0.010 | 0.015 | 0.006 | 0.003 | 0.015 | 0.015 | 0.015 | 0.013 | 0.003 | 0.008 | 0.016 | 0.003 |  |  |
| **H20** | 0.002 | 0.008 | 0.011 | 0.015 | 0.003 | 0.013 | 0.010 | 0.015 | 0.006 | 0.003 | 0.015 | 0.015 | 0.015 | 0.013 | 0.003 | 0.008 | 0.016 | 0.003 | 0.003 |  |
